# Building Interdisciplinary Research Capacity: a Key Challenge for Ecological Approaches in Public Health

**DOI:** 10.3934/publichealth.2016.2.389

**Published:** 2016-06-13

**Authors:** Lindsay P. Galway, Margot W. Parkes, Diana Allen, Tim K. Takaro

**Affiliations:** 1 Department of Health Sciences, Lakehead University, 955 Oliver Rd, Thunder Bay, ON P7B 5E1, Canada; 2 School of Health Sciences, University of Northern British Columbia, 3333 University Way Prince George, BC V2N 4Z9, Canada; 3 Department of Earth Sciences, Simon Fraser University, 8888 University Drive Burnaby, BC, V5A 1S6; 4 Faculty of Health Sciences, Simon Fraser University, 8888 University Drive Burnaby, BC, V5A 1S6

**Keywords:** interdisciplinary research, interdisciplinarity, ecological, public health

## Abstract

The shortcomings of public health research informed by reductionist and fragmented biomedical approaches and the emergence of wicked problems are fueling a renewed interest in ecological approaches in public health. Despite the central role of interdisciplinarity in the context of ecological approaches in public health research, inadequate attention has been given to the specific challenge of doing interdisciplinary research in practice. As a result, important knowledge gaps exist with regards to the practice of interdisciplinary research. We argue that explicit attention towards the challenge of doing interdisciplinary research is critical in order to effectively apply ecological approaches to public health issues. This paper draws on our experiences developing and conducting an interdisciplinary research project exploring the links among climate change, water, and health to highlight five specific insights which we see as relevant to building capacity for interdisciplinary research specifically, and which have particular relevance to addressing the integrative challenges demanded by ecological approaches to address public health issues. These lessons include: (i) the need for frameworks that facilitate integration; (ii) emphasize learning-by-doing; (iii) the benefits of examining issues at multiple scales; (iv) make the implicit, explicit; and (v) the need for reflective practice. By synthesizing and sharing experiences gained by engaging in interdisciplinary inquiries using an ecological approach, this paper responds to a growing need to build interdisciplinary research capacity as a means for advancing the ecological public health agenda more broadly.

## 1. Introduction

Many of the most pressing public health issues of the 21^st^ century are best described as ‘wicked problems’ [1]. Churchman coined this term in 1967 to describe public policy issues “that (1) are embedded in society; (2) display complex interdependencies that escape simple definition; (3) are not solvable by ‘taming’ or addressing ‘manageable’ sub-problems; and (4) often result in unintended consequences” [2]. Climate change, which has been called the greatest threat to public health of our time [3], is increasingly recognized as an example of a wicked problem [4]. At the same time, there is growing acknowledgement that fragmented, linear, and discipline-driven research as well as biomedical approaches are inadequate and ineffective for understanding and responding to wicked health problems like climate change [2],[4].

The application of ecological approaches in the public health context has emerged as a promising alternative to the traditional biomedical approach with particular relevance to wicked problems like climate change that span natural, social, and health systems and sciences [5]. Ecological approaches applied to public health issues acknowledge the role of social systems and ecosystems in the production of health, draw on complexity theory and systems thinking, and embrace context, uncertainty, and diversity. Following Charron we define an approach as “…a mindset that orients a process of inquiry” rather than as a methodology or framework [6]. Specific examples of the application of ecological approaches in the context of public health include: ecosystem approaches to health (ecohealth) [7],[8], One Health [9], and planetary health [10]. Taken together, these can be conceptualized as a “tapestry” [11] of emerging and interconnected approaches that seek to understand the complex linkages between ecosystems, social systems, ecological change, and human health and wellbeing.

Ecological approaches to public health have been described as “overtly interdisciplinary” [12] often requiring that researchers from a range of disciplines across the natural, social and health sciences learn and work together effectively [4],[13]–[16]. The application of ecological approaches demands scholars and practitioners “who can transcend disciplinary boundaries, work collaboratively, and handle complexity…” [17]. For decades, there have been widespread calls for interdisciplinary research within the ecological approaches literature, the public health literature, and beyond and the need for interdisciplinary research across the natural, social, and health sciences has been emphasized more recently [18],[19]. Despite the widespread calls for interdisciplinary research, relatively little is known about *how* to effectively and efficiently go about interdisciplinary research and how to resolve and respond to the unique challenges of doing interdisciplinary research [20]. We argue that limited progress will be made with the ecological public health agenda without acknowledging the challenges inherent in interdisciplinary research and without sharing lessons learned about navigating and resolving these challenges. To respond adequately to the increasingly calls for interdisciplinary research, additional and explicit attention must be given to the many challenges of doing interdisciplinary research in practice and to the challenge of building capacity for interdisciplinary research [20]. This paper responds to these challenges by identifying, synthesizing, and sharing lessons learned from doing interdisciplinary research applying an ecological approach at the intersection of climate change, water, and health. By synthesizing and sharing the major lessons that we have learned from our experiences navigating the challenges and complexities of interdisciplinary research, we hope that this paper will stimulate continued discussion about how we can and should go about interdisciplinary research, contribute toward building capacity for interdisciplinary research in practice, and ultimately enhance the application and effectiveness of ecological approaches in relation to public health problems.

**Figure 1.**
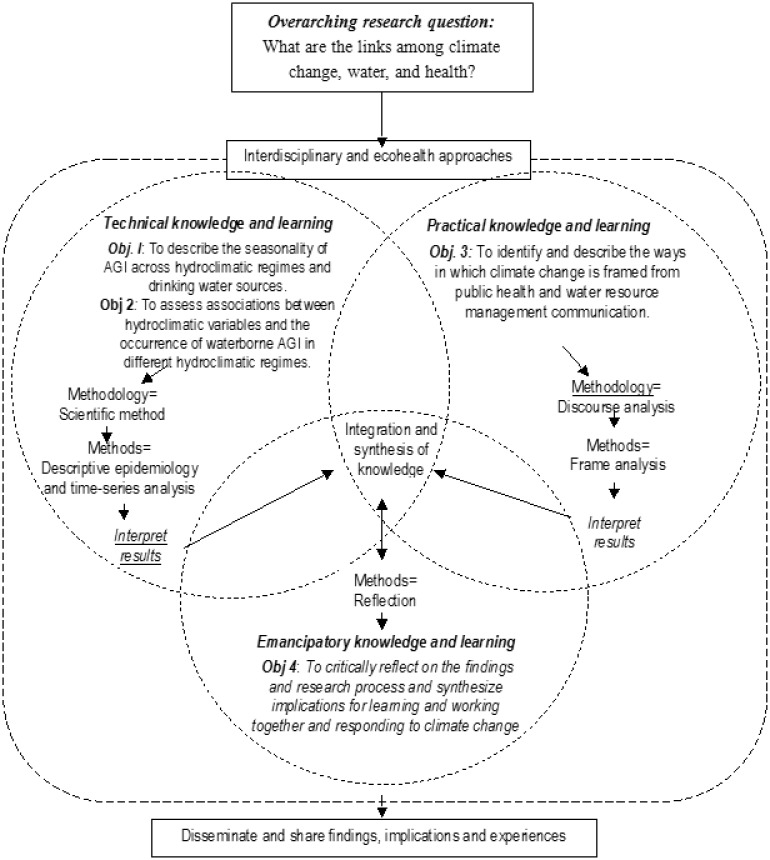
Overview of research process

**Figure 2.**
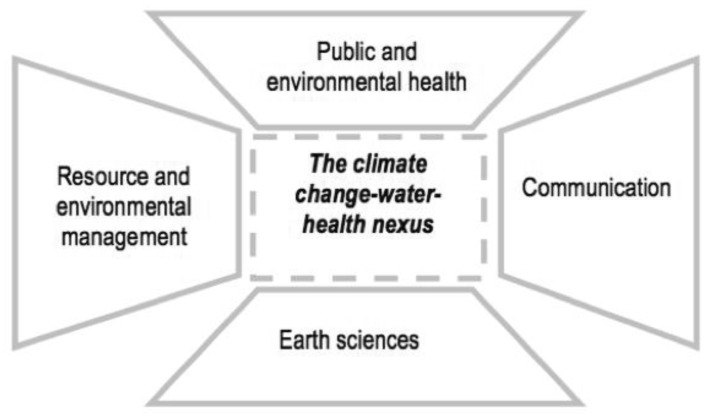
Main contributing disciplines

From 2011–2015, the authors engaged in an interdisciplinary research project guided by the following overarching research question: ‘what are the links among climate change, water, and health?’. We examined this question in the context of British Columbia Canada. Figure 1 provides an overview of the research process, as well as the specific research questions and research objectives that were pursued, and the methodologies and methods that were utilized throughout the process. The research was grounded in, and informed by an ecological approach [6]. The research was developed and conducted by a group of researchers representing the natural, social, and health sciences with a range of past experience with research that crosses disciplinary and sectoral boundaries(i.e.,[21]–[26]). The links among climate change, water, and health were explored by drawing on, and integrating, the literature, knowledge, and methods from the following disciplines in particular: public and environmental health, earth sciences, resource and environmental management, and communication (Figure 2). The research process resulted in three publications [27]–[29] which reflect the scope and the integrated nature of the research outputs.

Numerous challenges arose during our interdisciplinary research processes ranging from the need to manage disciplinary and divergent interpretations of the research problem to a lack of clarity about what exactly should be integrated. Reflecting on our experiences developing and conducting our interdisciplinary inquiry, this paper summarizes and presents specific lessons that our team learned, that proved useful for navigating and managing the challenges that arose, and which we believe will be helpful to other researchers engaging in interdisciplinary research applying an ecological approach to understand and address a wicked public health problem and working across the natural, social, and health sciences. As Lyall and Meagher argue, “the ability to anticipate potential challenges and troubleshoot problems early may help such project leaders to manage interdisciplinary research successfully”[30].We begin by briefly defining and characterizing interdisciplinary research in general. We then present and discuss the following five specific lessons: (i) the need for frameworks that facilitate integration; (ii) emphasize learning-by-doing; (iii) the benefits of examining issues at multiple scales; (iv) make the implicit, explicit; (v) the need for reflective practice.

## 2. Defining and characterizing interdisciplinary research

In the literature, definitions for interdisciplinary research abound and debates about what qualifies as interdisciplinarity endure [31]. Nevertheless, key characteristics of interdisciplinary research can be identified around which consensus is developing. Integration is a key feature of interdisciplinary research, and is generally seen as the product or goal of interdisciplinary research [32],[33]. Integration is a process whereby ideas, data, knowledge, methods, concepts, and/or theories are combined to develop a more “comprehensive understanding of the research problem than any one discipline could develop alone”[34]. Ultimately, integration produces an output that is greater than the sum of its parts resulting in emergent understanding that is well-suited to the complexity of wicked problems [2],[35],[36]. Along a similar vein, McDonell has argued that, when engaging in interdisciplinary research, “disciplines collaborate in such a way that each takes up some of the assumptions and worldviews and languages of the others” [37]. Another key characteristic of interdisciplinary research is a focus on generating useful knowledge; i.e., knowledge that can be used to inform policy and action and therefor contributes towards addressing the issue at hand. This is highlighted by Lemos and Morehouse, who define interdisciplinary research as the effort of actors “from different disciplines to work together to tackle problems whose solutions cannot be achieved by any single discipline” [38]. A third key characteristic of interdisciplinary research is the central role of diversity; interdisciplinary research welcomes and values diversity in terms of ways of knowing, seeing, and doing. The value of this diversity is especially pertinent when addressing wicked problems, that span scales and perspectives and cannot be adequately understood or addressed from any single perspective [8].

A further important characteristic is that there is no single, or even dominant, interdisciplinary research methodology; instead, a suite of diverse methodologies and methods are employed when conducting interdisciplinary inquiries. Rather than relying on disciplinary traditions alone, the research problem and research questions at hand guide methodological decisions and thereby, the interdisciplinary research process [15],[39]. Taking these central characteristics together, interdisciplinary research can be understood as an iterative process aimed at generating useful knowledge in relation to wicked problems through integration and drawing on diversity in terms of knowledge, perspectives, and research methods.

Two additional trends emerge from the recent literature on interdisciplinary research and interdisciplinarity. First, the literature largely acknowledges that interdisciplinary research is distinct from multi- and transdisciplinary research which have also been proposed as approaches to overcome the limitations of disciplinary boundaries when seeking to address complex contemporary public health problems [2],[4],[16]. Multidisciplinary research utilizes knowledge from different disciplines, but integration is not an explicit aim and often remains limited in practice. Max-Neef describes multi-disciplinary research in the following manner: “members carry out their analyses separately, as seen from the perspective of their individual disciplines, the final result being a series of reports pasted together, without any integrating synthesis” [40]. Transdisciplinary research seeks integration across disciplinary and sectoral divides, but also includes non-academic perspectives and knowledge and prioritizes community participation and engagement throughout the research process [6]. Transdisciplinary research therefore seeks integration across ‘knowledge cultures’ [4] drawing on participatory and community-based research methodologies [41]

The notion of scope is a second noteworthy aspect emerging from the recent literature on interdisciplinary research and interdisciplinarity [19],[42]. As an example, Huutoniemi et al. distinguish between ‘narrow’ and ‘broad’ interdisciplinary research in the following ways:

*In narrow interdisciplinarity, participating fields are conceptually close to each other…The interaction between fields is not exceptional or particularly challenging in epistemological terms since the concepts, theories and/or methods are relatively similar in their epistemological presuppositions. The ingredients of broad interdisciplinarity, in contrast, originate from conceptually diverse fields that cross the boundaries of broad intellectual areas (e.g. law and engineering, cultural studies and medicine, philology and neurology). In these projects, advanced interaction may become a real challenge because of the epistemological heterogeneity and thus increase the likelihood of conflict and shortfalls of integration* [42]. Similar ideas can be seen in descriptions of the scope of interdisciplinarity research using terms such as small versus big [43] or deep versus shallow [19].

Public health research seeking to employ an ecological approach by drawing on ideas, concepts, methods, and methodologies across the natural, social, and health sciences provides a clear example of ‘broad’ interdisciplinarity [4],[14]. Generally speaking, numerous challenges and obstacles arise when engaging in interdisciplinary research processes. ‘Broad’ interdisciplinary research is particularly challenging as it demands the management of wide ranging assumptions, vocabularies, and priorities, while also navigating important, and sometimes seemingly intractable differences in terms of epistemology, ontology, and methodological orientations that characterizes distinct disciplinary traditions.

## 3. Insights for building interdisciplinary research capacity

This section presents the five specific challenges and lessons that arose during the interdisciplinary inquiry. Table 1 provides an overview of these features, and a point of reference for others seeking to anticipate and respond to these challenges in the design and conduct of interdisciplinary research.

**Table 1.** Summary of challenges and lessons learned from an interdisciplinary inquiry at the intersection of climate change, water, and health.

Challenge that arose	Lesson(s) learned to navigate challenge
Disciplinary interpretation and understanding of the research question/issue	The need for reflective practice
Different disciplines interested in/focused on different scales	Zooming in, and zooming out
Negotiating unique languages, vocabularies, epistemologies, methodological orientations underlying different disciplinary traditions	Make the implicit, explicit
Lack of clarity about what should be/could be integrated	The need for frameworks that facilitate integration
Difficulties cultivating shared agreement on research methods	Emphasize learning-by-doing

### 3.1 The need for frameworks that promote integration

As described above, integration is a defining feature of interdisciplinary research. Despite the central role of integration in interdisciplinary research, it remains a major conceptual and methodological challenge. Frameworks, defined here as “tools to think with” [44], are needed to promote and facilitate integration. Although frameworks have been proposed to facilitate interdisciplinary research (many of which have emerged from the field of natural resource management and conservation i.e., [45] ) , frameworks that promote ‘broad’ interdisciplinarity and integration across the natural, social, and health sciences are rare despite being particularly relevant to the effective application of ecological approaches [46].

Our research at the intersection of climate change, water, and health drew on Habermas' *Theory of Knowledge and Human Interests* to develop a framework to guide and promote integration and the generation of integrated research outputs and learning. Habermas' *Theory of Knowledge and Human Interests* describes three basic human interests which leads to three domains of knowledge: technical knowledge, practical knowledge, and emancipatory knowledge [47]. According to Habermas, human interest in managing and controlling our environment leads to the pursuit of technical knowledge. Technical knowledge is focused on cause-effect relationships and is empirically derived. Technical knowledge aims to explain a phenomenon or issue of interest. Practical knowledge, Habermas' second knowledge domain, is socially constructed and relevant for policy and practice [47]. Practical knowledge emerges because humans have an interest “in living together in a society and coordinating social actions …we therefore need to understand each other, both on a simple personal level and on a larger social and political level” [48]. Practical knowledge is focused on “… the understanding of ourselves, others, and the social norms of the community or society in which we live” [48]. Emancipatory knowledge, the third knowledge domain, emerges from the human drive for personal growth, which “can lead us to critically question assumptions, values, beliefs, norms, and perspectives” [48]. It is only through the production of emancipatory knowledge that we are able to identify and account for values, interests, and power structures, and thereby legitimize technical and practical knowledge [4],[47]. In developing this framework, we noted clear parallels between the three domains of knowledge presented by Habermas and the three major research paradigms: the positivist, constructivist, and critical paradigms.

**Figure 3.**
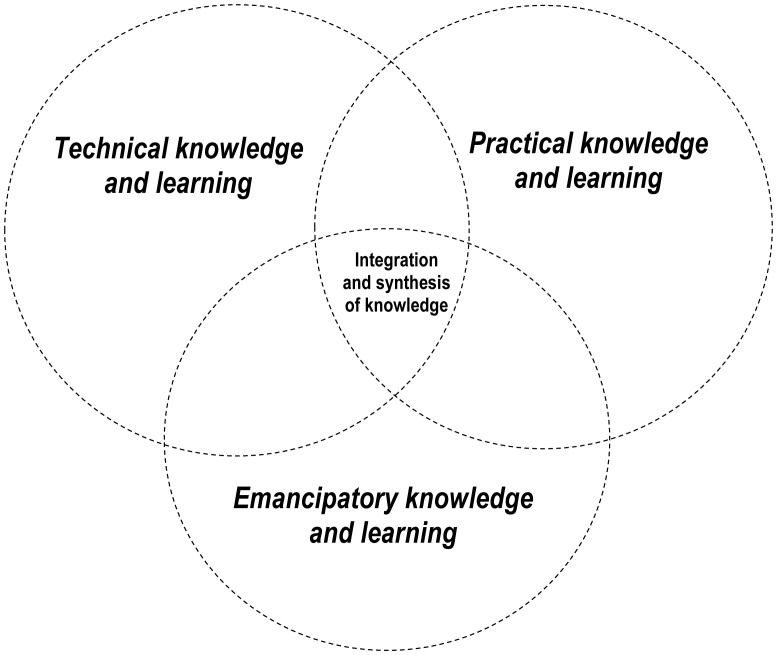
The Three Domains of Knowledge and Learning Framework (adapted from [48]).

Drawing on Habermas' theory, the framework we designed for promoting integration in our research was entitled the *Three Domains of Knowledge and Learning Framework* (see Figure 3). Figure 1 illustrates the connections between the *Three Domains of Knowledge and Learning Framework*, the research questions and objectives, and the methodologies and methods.

Although seemingly simple, the focus on knowledge domains within the framework, rather than traditional disciplinary perspectives and boundaries, encourages ‘broad’ interdisciplinarity across multiple “conceptually diverse” [42] disciplines characterized by distinct epistemologies, ontologies, and methodologies. Another feature of the *Three Domains of Knowledge and Learning Framework* with relevance to interdisciplinary research is that it makes explicit that each of the different types of knowledge and different ways of knowing (i.e., technical, practical, and emancipatory) are valid and valuable contributions towards generating integrated understanding of wicked problems. This contrasts the common situation – in public health research and many other applied fields – where technical knowledge is prioritized and seen as more valuable relative to others forms of knowledge, such that practical knowledge, and more so, emancipatory knowledge, tend to be overlooked [49]. The framework we propose (Figure 3) addresses this issue by presenting each of the three knowledge domains as equally relevant and valid in pursuit of integrated knowledge of a phenomena or issue under examining. An additional strength of the *Three Domains of Knowledge and Learning Framework* is that it makes a clear distinction between *knowledge* and *learning*. This distinction is often overlooked in research activities. Learning, which we understand as a shift in perspective inspired by reflection on experiences is distinct from knowledge generation. Learning can be instrumental in finding common ground and inspiring innovation to overcome barriers that tend to impede progress in interdisciplinary research processes.

In our research, the *Three Domains of Knowledge and Learning Framework* facilitated integration across the disciplinary perspectives outlined in Figure 2 as well as the natural, social, and health sciences more broadly. Integration across these divides, is a common challenge when applying an ecological approach to public health issues. The research outputs reported in [27]–[29] are illustrative of the integrated nature of the knowledge generated and showcase the benefits of ‘broad’ interdisciplinarity across the natural, social, and health sciences enabling a more fulsome understanding of the links among climate change, water, and health than could have been achieved using any one disciplinary perspective. As an example, to achieve our first and second research objectives (see Figure 1), we assessed the seasonal trends and ecological drivers of waterborne gastro-intestinal illness in study communities selected to represent two dominant hydroclimatic regimes: snowmelt-dominated and rainfall-dominated regimes. Specifically, study communities were selected to represent either snowmelt- or rainfall-dominated watersheds. Although hydroclimatology, i.e. the dominant climatic drivers for watershed responses [50] is a construct that is commonly applied in the natual sciences, it is rarely applied to advance our understanding of health outcomes. Moreover, to date, nearly all research examining the links between climate change and water-related health outcomes have been “organized spatially around human constructs” [51] rather than being organized around constructs that are relevant to the natural sciences such as watersheds. Results from our work illustrated distinct trends and relationships in the context of a snowmelt-dominated versus rainfall-dominated watershed regimes underscoring the value of applying the construct of hydroclimatic regimes to understand the complex linkages between climate change, water, and human health [27],[28]. This knowledge is a novel contribution that could not have been achieved in the absence of interdisciplinarity. A further example of the integrated research outputs emerging from our interdisciplinary research is our paper [29]. This study used frame analysis, a method commonly employed in the social sciences, to examine and summarize the ways in which climate change is constructed and understood in the public health and water resource management sectors. Framing is a social process that involves the “selection and salience” [52] of different aspects of an issue, prioritizing certain responses or solutions, and drawing on different rationale to mobilize action [52], [53]. Effective climate change policy and action requires explicit attention to the ways in which climate change is framed [54]. Our analyses showed that there are numerous frames for climate change within the public health and water resource management sectors and that exploring framing similarities and differences can highlight constraints and enabling factors for inter-sectoral adaptation [29].

In summary, our experiences have shown that the *Three Domains of Knowledge and Learning Framework* stimulated diverse ways of thinking about the climate change-water-health nexus, encouraged methodological diversity, and inspired learning which together resulted in integrated research outputs and a more fulsome understanding of the linkages among climate change-water- and health than would have been achieved using disciplinary research.

### 3.2 Emphasize learning-by-doing

Our experiences doing research at the intersection of climate change, water, and health have underscored the value of “learning-by-doing” i.e., an iterative process of learning from experience, in interdisciplinary research processes [55]. Learning-by-doing is emphasized in certain research methodologies (e.g., action-research) and fields (i.e., natural resource management) but it is not explicitly emphasized nor promoted in relation to interdisciplinary research or ecological approaches in public health. Because there is no single model or roadmap for success when it comes to interdisciplinary research and the application of ecological approaches in public health, researchers and research teams stand to benefit from explicitly incorporating their learning in an iterative manner throughout the research process [8]. In other words, when dealing with complex, wicked problems that are poorly understood by single disciplines, there is much to be gained from moving forward in the process of knowledge generation in an iterative manner, drawing on lessons learned and insight gained along the way, and modifying objectives, methods, and analytical procedures that reflect a learning-by-doing approach.

### 3.3 The benefits of examining research questions at multiple scales - zooming in, and zooming out

The value of examining research questions across multiple scales, i.e., ‘zooming in and zooming out’ in relation to the study topic(s), became evident over the course of our research activities. We learned that by considering the links between climate change, water, and health at different scales, individual person, community, watershed, and provincial scales in our case, “…new properties emerge into view” [56]. Waltner-Toews has argued that examining research questions at multiple scales, or zooming in and out, is an essential ‘imaginative skill’ for generating a more fulsome and contextualized understanding of wicked problems [56], an idea which resonates strongly with what Brown et al. describe as the critical importance of imagination when seeking to address wicked problems [3]. An added benefit of zooming in and out in the context of interdisciplinary research is that this process can enable the identification of cross-scalar connectedness and relationships which tend to be overlooked but can be very relevant to informing multi-level policy and action. Moreover, we also realized that it is useful to think about the process of zooming in and zooming out in relation to integration. Building on the works of Parkes et al. [36],[41],[57], which present ‘horizontal’ and ‘vertical’ integration as two types of integration, we present ‘scalar integration’ across scales as a third type of integration. Explicitly exploring research questions and synthesizing knowledge across multiple scales of analyses is a distinct form of integration, and warrants further attention as a central facet of ecological approaches to public health problems that reflect interdisciplinarity, systems thinking, and seek to embrace context, uncertainty, and diversity. It is worth noting here that although we identified meaningful benefits from explicitly considering our research question (i.e., ‘what are the links among climate change, water, and health?’) at multiple scales, cross-scalar examination can generate unique analytical and conceptual challenges.

### 3.4 Make the implicit, explicit

The themes of communication and language are frequently cited as key challenges in interdisciplinary research processes [58]. We found this to be the case in our interdisciplinary and ecologically oriented public health inquiry. Disciplines and sectors have their own language and terminology, their own understanding of concepts, and their own sets of assumptions, priorities, and biases. In our research, however, we realized that rather than focusing on communication and language as barriers or challenges, progress could be made by using communication and language as tools to make the implicit, explicit and to ultimately facilitate knowledge sharing, integration, and collaboration throughout interdisciplinary research processes.

As an example, our research at the intersection of climate change, water, and health, identified problem framing as an important tool to facilitate intersectoral collaboration in relation to climate change policy and action [29]. Upon further reflection, we acknowledged the influence of problem framing in our own research efforts and came to realize the potential of problem framing as a tool to make the implicit, explicit within our interdisciplinary team. As mentioned above, framing is a social process that involves the “selection and salience” [52] of different aspects of an issue. When distinct problem frames are left implicit and unacknowledged, this can impede mutual understanding and integration, ultimately delaying or impeding progress in interdisciplinary research [59]–[61]. By discussing problem framing, research team members can expose underlying assumptions, priorities, and biases that are all too often left unspoken [62]. Purposeful attention to problem framing is particularly relevant in relation to wicked problems “wherein stakeholders may have conflicting interpretations of the problem and the science behind it” [63]. We also realized that, having made framing differences explicit in the context of interdisciplinary work, it is important not to attempt to do away with framing differences, or to establish that one particular way of framing a given research problem is better than another. Tendencies to ignore or trump alternative frames work against the aim of effectively learning and working together to generate integrated knowledge about wicked problems [64]–[66]. Instead, the task at hand is to explore problem framing in a constructive manner, to acknowledge different ways of understanding and conceptualizing a given research problem, and to embrace the diversity of problem frames that inevitably emerge during interdisciplinary research processes [67].

Informed by the insights from this research, we propose that this can be achieved by allocating time throughout the research process, particularly in the early stages, to identify points of convergence and divergence regarding problem framing. More specifically, identifying points of divergence that may impede integration and inhibit progress while identifying points of overlap that can promote and motivate progress [68]. Depending on the relationships among research team members and the structure of the team, the influence of power and hierarchy may need to be acknowledged and addressed while collaboratively exploring problem framing. In short, purposeful attention to problem framing can be a simple but effective communication tool to make the implicit, explicit and thereby help us move beyond disciplinary silos and reductionist approaches towards integration and interdisciplinarity.

### 3.5 The need for reflective practice

Particular challenges emerge when engaging in interdisciplinary research. Consequently, an appropriate set of tools and strategies are needed to navigate the complexity, messiness, and uncertainty that characterizes boundary-crossing and integrative work [36],[57],[69]. Reflection and reflective practice should be acknowledged as particularly valuable tools in the interdisciplinary researchers toolbox [70]–[72]. According to Dewey, “[t]o reflect is to look back over what has been done so as to extract the net meanings which are the capital stock for intelligent dealing with further experiences. It is the heart of intellectual organization and of the disciplined mind” [73]. Reflection is “a systematic, rigorous, disciplined way of thinking, with its roots in scientific inquiry” [74]. Reflection can take many forms “[i]t can be an individual or group activity; it can be formative, cumulative or summative; verbal or written; shared or introspective; assessed or non-assessed” [75]. Reflection can help us move beyond disconnected and fragmented knowledge and information towards shared understanding and integration [76]. Cornell goes as far as to say that researchers simply cannot integrate knowledge without engaging in the process of reflection [71]. While Romm (1998) suggests that interdisciplinarity can be understood as “embracing a reflexive orientation” [70].

Our experiences have underscored that reflective practice throughout the research process helps to accommodate and manage different perspective and epistemologies involved in interdisciplinary inquiries and facilitate effective dialogue on problem framing, while also fostering the creativity, innovation, and imagination that are often needed to navigate the unique challenges that emerge during interdisciplinary research, especially in the interdisciplinary terrain where natural, social, and health systems and sciences intersect [2],[3],[50]. It should be noted that engaging in reflection and reflective practice can unearth power imbalances, conflicts, or differences in priorities [78]. Discussing and working through any such issues that arise in a collaborative and respectful manner can help to move the research team to appreciate the various skills, bodies of knowledge, and perspectives that the various team members bring to the project and ultimately help to move the project forward.

Researchers engaging in interdisciplinary inquiries applying an ecological approach stand to benefit from building skills in reflection and becoming what Schön calls “reflective practitioners” [79]. In his seminal and highly influential book entitled *The Reflective Practitioner*, Schön [79] describes a reflective practitioner in the following manner: *The [reflective] practitioner allows himself (sic) to experience surprise, puzzlement, or confusion in a situation, which he finds uncertain or unique. He (sic) reflects on the phenomenon before him, and on the prior understandings, which have been implicit in his behavior. He (sic) carries out an experiment, which serves to generate both a new understanding of the phenomenon and a change in the situation.* [79]

**Figure 4.**
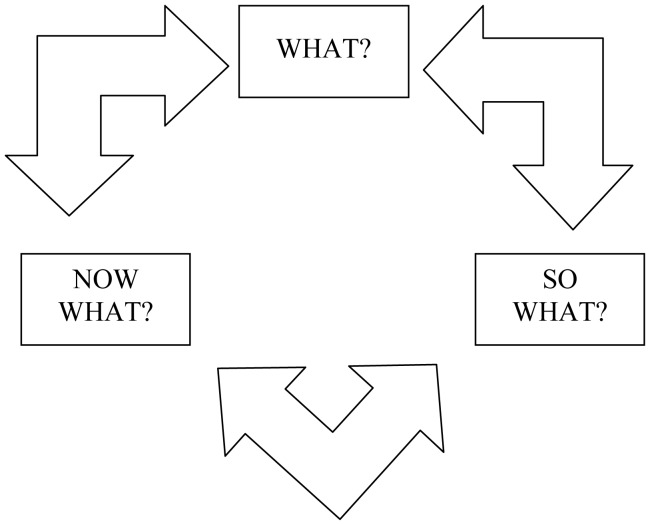
Framework for reflective practice (adapted from [50])

Despite the numerous benefits of reflection and reflective practice, many researchers are generally unhabituated to reflection and lack the skills to engage in reflective practice [71]. Developing the capacity for reflective practice may therefore need to be purposefully addressed within interdisciplinary research by drawing on specific design tools, frameworks, and strategies that foster reflection (e.g., [4],[80],[81].) In our research, reflective pauses where taken frequently throughout the research process. We utilized Rolfe et al.'s [81] *Framework for Reflective Practice* [81] (see Figure 4) as a tool to encourage reflection and learning. Rolfe et al.'s framework presents a cycle consisting of the three simple questions: 1) *What*?; 2) *So what*?; and 3) *Now what*? Taking the time to consider and discuss these simple questions throughout a research process can encourage reflective practice and can generate mutual respect as well as shared language and understanding between research team members. This can be done either individually or collaborative. An additional effective and simple tool for promoting reflective practice in research is keeping a reflective research journal [82]. Reflective journals provide an “opportunity to capture reflective insights” and a space for individuals to reflect on new ideas, concepts, and theories and to work through some of the ontological, epistemological, and methodological challenges and conflicts that arise in interdisciplinary research [75]. From the perspective of the lead author in the context of our research exploring the links among climate change, water, and health, keeping a reflective research journal enabled the identification of biases and provided a safe space to explore alternative ways of thinking and framing the research problem. Emerging interdisciplinary scholars and interdisciplinary scholars in training may, in particular, benefit from the practice keeping a reflective research journal.

## 4. Conclusion

The need for research that is explicitly focused on understanding the complex, interconnected, and wide-reaching impacts of ecosystems and ecosystems change on human health and well-being is increasingly apparent [83]. The application of ecological approaches, which are characteristically interdisciplinary, is one of our best options for understanding complex linkages, addressing wicked problems, and making progress towards human and planetary health [84]. Now is the time to head the calls “…to do more interdisciplinary research and to do it better” [85]. The influential anthropologist Clifford Geertz has suggested that the scholarly trend towards interdisciplinarity is not just “the moving of a few disputed borders, the marking of some more picturesque mountain lakes - but an alteration of the principles of mapping. Something is happening to the way we think about the way we think” [86]. Clearly, altering the way we think about the way we think is no easy task.

We argue that explicit attention towards the challenge of doing interdisciplinary research is critical in order to effectively apply ecological approaches to public health issues. In an effort to address the interdisciplinary research imperative, we have outlined specific opportunities for addressing certain common challenges of interdisciplinary research and for building interdisciplinary research capacity with particular relevance to ecological approaches in public health by drawing on our experiences conducting interdisciplinary research at the interface of climate change, water, and health.
